# Rapid microevolution of biofilm cells in response to antibiotics

**DOI:** 10.1038/s41522-019-0108-3

**Published:** 2019-11-06

**Authors:** Anahit Penesyan, Stephanie S. Nagy, Staffan Kjelleberg, Michael R. Gillings, Ian T. Paulsen

**Affiliations:** 1 Department of Molecular Sciences, Faculty of Science and Engineering, Macquarie University, Sydney, NSW 2109 Australia; 2 School of Chemical Engineering, University of New South Wales, Sydney, NSW 2052 Australia; 3 Singapore Centre for Environmental Life Sciences Engineering, 60 Nanyang Drive, SBS-01N-27, Singapore, 637551 Singapore; 4 School of Biological Sciences, Nanyang Technological University, 60 Nanyang Drive, Singapore, 637551 Singapore; 5 School of Biological, Earth and Environmental Sciences, University of New South Wales, Sydney, NSW 2052 Australia; 6 Department of Biological Sciences, Faculty of Science and Engineering, Macquarie University, Sydney, NSW 2109 Australia

**Keywords:** Biofilms, Antimicrobials

## Abstract

Infections caused by *Acinetobacter baumannii* are increasingly antibiotic resistant, generating a significant public health problem. Like many bacteria, *A. baumannii* adopts a biofilm lifestyle that enhances its antibiotic resistance and environmental resilience. Biofilms represent the predominant mode of microbial life, but research into antibiotic resistance has mainly focused on planktonic cells. We investigated the dynamics of *A. baumannii* biofilms in the presence of antibiotics. A 3-day exposure of *A. baumannii* biofilms to sub-inhibitory concentrations of antibiotics had a profound effect, increasing biofilm formation and antibiotic resistance in the majority of biofilm dispersal isolates. Cells dispersing from biofilms were genome sequenced to identify mutations accumulating in their genomes, and network analysis linked these mutations to their phenotypes. Transcriptomics of biofilms confirmed the network analysis results, revealing novel gene functions of relevance to both resistance and biofilm formation. This approach is a rapid and objective tool for investigating resistance dynamics of biofilms.

## Introduction

*Acinetobacter baumannii* is a Gram-negative pathogen found in hospitals worldwide.^1^ It is responsible for opportunistic infections of the bloodstream, urinary tract, and other soft tissues, and can account for up to 20% of infections in Intensive Care Units, causing serious morbidity and mortality.^1,2^ *Acinetobacter baumannii* belongs to a group of six pathogens responsible for many multidrug-resistant (MDR) nosocomial infections (the ESKAPE pathogens: *Enterococcus faecium*, *Staphylococcus aureus*, *Klebsiella pneumoniae*, *Acinetobacter baumannii*, *Pseudomonas aeruginosa*, and *Enterobacter* spp.).^3^ In 2017, *A. baumannii* was listed at the top of the highest priority “Critical” group of antibiotic-resistant pathogens identified by the World Health Organization as in need of further research.^4^

The success of this pathogen is due to a combination of cellular resistance mechanisms and the additional protection provided by its biofilm lifestyle.^5^ *Acinetobacter baumannii* has an arsenal of tools to defend against antimicrobials, including classical mechanisms of antibiotic resistance, such as enzymatic inactivation of antibiotics, target and membrane modifications, and active export of drugs via membrane-localized drug efflux transporters.^6–8^ These mechanisms have been extensively studied in planktonically grown *A. baumannii*. Transcriptomic and mutational studies under antibiotic challenge using *A. baumannii* planktonic cultures identified mutations and differentially expressed genes directly linked with these known mechanisms of resistance to particular antibiotics.^9,10^

Resistance and pathogenicity of *A. baumannii* is enhanced by its ability to form biofilms.^5^ *Acinetobacter baumannii* biofilms can form on various surfaces, including medical devices, where they are persistent sources of contamination and infection.^11^ The National Institutes of Health (NIH) estimates that biofilms account for over 80% of microbial infections in the body.^12^ Biofilms are a major obstacle to treatment because their cells can display up to a 1000-fold increase in antibiotic resistance compared to planktonic cells.^13,14^ Biofilms provide additional resistance^5^ (also referred to as “biofilm tolerance”^15–17^) via biofilm-specific mechanisms such as the shielding effect of the biofilm matrix that leads to restricted penetration of antimicrobials,^18^ the slower growth rate in deep layers of biofilms,^19^ and the presence of persister cells.^20^

Biofilms are recognized as the predominant form of bacterial life, with the majority of bacteria living as biofilm communities in diverse environments, including within host organisms.^15,21^ Nevertheless, compared to the wealth of data collected using planktonic cultures during the history of microbiological research, the biofilm mode of life remains largely underexplored, leading to a growing interest in the ecology of microbial biofilms, and the factors involved in biofilm development and survival. In particular, understanding the processes that occur in biofilms when exposed to antibiotics is important, because this could give us insight into how advantageous phenotypes arise, and help identify the genomic basis of these phenotypes.

The ability of bacteria to adapt to new environmental conditions arises from their short generation times and genomic variability, allowing rapid emergence of favorable mutations. Methods for investigating the effect of mutations on bacterial phenotype, such as knock-out strains and transposon mutagenesis, are often time-consuming, involve extensive sample manipulation, and often focus on single gene targets. In contrast, whole genome sequencing can rapidly identify suites of naturally occurring mutations, and also reveal potential synergy between different mutations.

This study investigated how genetic and phenotypic diversity was generated within biofilms of a highly virulent strain of *A. baumannii*. We assessed the transcription profiles of biofilms grown in the presence and absence of sub-inhibitory concentration of antibiotics ciprofloxacin and tetracycline, and examined the genetic consequences of biofilm growth in the presence of antibiotics, identifying de novo mutations by using whole genome sequencing. Thus, we were able to link phenotypes with genotypes and with population level gene expression patterns in one experimental analysis, providing a holistic assessment of processes that occur in biofilms, and, subsequently, drive genomic changes under the exposure to sub-inhibitory concentration of antibiotics.

To assess changes in antibiotic susceptibility, the MIC (minimum inhibitory concentration) levels of biofilm dispersal cells were assessed against the antibiotics in a broth microdilution assay, and compared to the MIC levels of initial planktonic cultures. The MIC broth microdilution assay tests the level of antibiotic resistance in the planktonic state, that is, the level of resistance determined by classical “cellular” antibiotic resistance mechanisms^5^ and does not capture the additional resistance provided by biofilm mode of life. Additional resistance provided by the biofilm lifestyle (“biofilm tolerance”) is directly related to the ability to form biofilms,^5^ which was tested separately. Thus, by using the broth microdilution MIC assay (to test “cellular” level resistance), and biofilm formation assays (to assess the ability to form biofilms), we were able to separate these two phenotypes, both of which can significantly contribute to the overall resilience of biofilms.

*Acinetobacter baumannii* is intrinsically resistant to many antibiotics, and consequently ciprofloxacin and tetracycline were used in this study, due to the relatively low level of resistance of *A. baumannii* AB5075-UW to these two antibiotics. These antibiotics are chemically diverse and belong to different classes, with different modes of actions. Ciprofloxacin (fluoroquinolone) functions via inhibiting DNA gyrase and topoisomerases involved in transcription, thereby inhibiting cell division.^22^ Tetracycline (tetracycline class) inhibits protein synthesis by binding to the 30S subunit of microbial ribosomes.^23^ Earlier studies also demonstrated that both ciprofloxacin and tetracycline are able to permeate biofilms,^18,24^ which is important to maximize the exposure of biofilm cells to antibiotics.

## Results and discussion

Understanding the dynamics of biofilms exposed to antibiotics is important for developing control strategies and for tracking the evolution of resistance. With this in mind, we exposed biofilms of a highly virulent clinical strain of *A. baumannii*, AB5075-UW, to sub-inhibitory concentrations of two antibiotics: ciprofloxacin and tetracycline. Phenotypic and genomic analyses were undertaken on cells dispersing from biofilms, while the biofilms were investigated using transcriptomics. This multipronged approach examined processes occurring in biofilm communities, reflected in differential gene expression, while simultaneously examining genomic changes in cells dispersing from biofilms.

### Ability to form biofilms

**Fig. 1:**
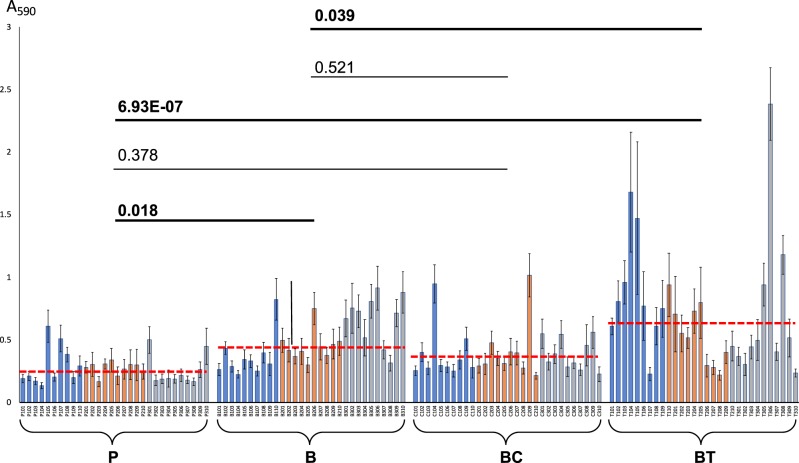
Results of biofilm formation assay. *X*-axis: 30 planktonic isolates P101–P310 (P samples), 30 antibiotic-free biofilm effluent isolates B101–B310 (B samples), 30 ciprofloxacin-exposed biofilm effluent isolates C101–C310 (BC samples), and 30 tetracycline-exposed biofilm effluent isolates T101–T310 (BT samples). Each sample type includes 10 isolates from each biological replicate 1 (blue bars), biological replicate 2 (orange bars), and biological replicate 3 (gray bars). The *Y*-axis represents corresponding absorbance values of 5-fold diluted crystal violet extracts at 590 nm (*A*_590_). Error bars represent standard deviations between 24 technical replicates. The average value for each sample type is indicated by red dashed lines. *P* values denote differences between sample pairs based on nested mixed-factor ANOVA test followed by Turkey’s HSD post hoc test. *P* values showing statistically significant (*p* < 0.05) differences are presented in bold

Biofilm formation can increase the resistance/tolerance of biofilms by orders of magnitude. Therefore, the ability to form biofilms has serious implications for antibiotic therapy. Biofilm formation was assessed using spectrophotometric quantification of biofilms stained with crystal violet (CV). Compared to the initial planktonic isolates, biofilm effluent isolates (with or without antibiotic exposure) showed increased biofilm formation capability (Fig. 1). Many tetracycline-exposed biofilm effluent isolates showed an additional increase in biofilm formation. Increased biofilm formation in the presence of tetracycline has been reported in other bacteria^25,26^ and can complicate treatment of biofilm-related infections, because the treatment itself promotes biofilm formation and enhances biofilm-specific resistance mechanisms (also known as “biofilm tolerance”).^5^ Here we demonstrate that these phenotypes can be fixed within populations of biofilm dispersal cells, which then display enhanced biofilm formation even after treatment has ceased.

Cells from ciprofloxacin-exposed biofilms showed no net change in biofilm formation in comparison to those from antibiotic-free biofilms and from planktonic cultures (Fig. 1). Some individual isolates from the ciprofloxacin and tetracycline treatments were outliers, exhibiting 5 to 10 times higher biofilm formation than planktonic cells (Fig. 1). This strongly suggested the accumulation of mutation(s) that enhanced biofilm formation.

### Evolution of antibiotic resistance

**Fig. 2:**
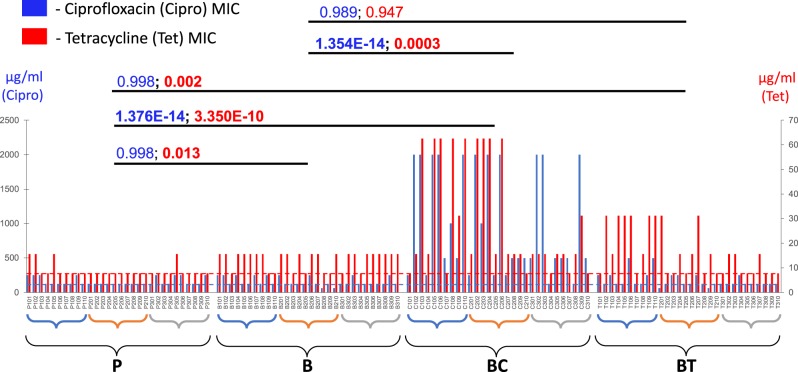
Results of the minimum inhibitory concentration (MIC) antibiotic susceptibility assay. *X*-axis: 30 planktonic isolates P101–P310 (P samples), 30 antibiotic-free biofilm effluent isolates B101–B310 (B samples), 30 ciprofloxacin-exposed biofilm effluent isolates C101–C310 (BC samples), and 30 tetracycline-exposed biofilm effluent isolates T101–T310 (BT samples). Each sample type includes 10 isolates from each biological replicate 1 (blue parentheses), biological replicate 2 (orange parentheses), and biological replicate 3 (gray parentheses). Blue bars represent the MIC levels of ciprofloxacin (in μg/ml, measured on the primary *Y*-axis on the left), and red bars—the MIC levels of tetracycline (in μg/ml, measured on the right-hand *Y-*axis). Consensus MIC levels in the initial planktonic cultures are shown by the horizontal blue (for ciprofloxacin MIC) and red (for tetracycline MIC) dashed lines. *P* values denote differences between sample pairs based on nested mixed factor ANOVA test followed by Turkey’s HSD post hoc test. *P* values showing statistically significant (*p* < 0.05) differences are presented in bold

Cells recovered from antibiotic-exposed biofilms showed consistent increases in antibiotic resistance in the MIC assay, above their initial MICs (Fig. 2). Out of 30 random isolates recovered from ciprofloxacin-exposed biofilms, the overwhelming majority (93%) showed increased resistance towards ciprofloxacin (2-fold or more increase in the MIC), with most (76%) showing at least a 4-fold increase in ciprofloxacin resistance. Many ciprofloxacin-exposed isolates (80%) also showed increased resistance to tetracycline, with one-third of the isolates showing high levels of resistance (4-fold and above increase in the MIC) (Fig. 2).

Similar effects were also observed in cells from tetracycline-exposed biofilms. More than half (53%) of the isolates displayed at least a 2-fold increase in resistance to tetracycline, with eight exhibiting high-level resistance, at 4-fold increases in the MIC or more. Nine of the tetracycline-exposed isolates also gained increased resistance to ciprofloxacin, in some cases exhibiting a 4-fold increase in the MIC. However, the net difference in average ciprofloxacin MIC levels between the set of 30 tetracycline-exposed isolates and the initial planktonic isolates was not statistically significant (Fig. 2), suggesting that despite an increased ciprofloxacin resistance observed in several isolates, tetracycline does not lead to a significant net increase in cross-resistance towards ciprofloxacin. In contrast, exposure of biofilms to ciprofloxacin led to a statistically significant net increase in the MIC levels for both antibiotics in cells dispersed from these biofilms (Fig. 2).

To further investigate whether biofilm-derived isolates gained cross-resistance against other antibiotics, the isolates were tested for their susceptibility to colistin and erythromycin, both of which differ structurally and functionally from antibiotics to which the biofilms were exposed. Our choice of antibiotics was limited due to the innate resistance of AB5075-UW to a broad range of antibiotics. Many of the isolates that showed increased resistance towards ciprofloxacin and/or tetracycline were also resistant towards erythromycin (Supplementary Fig. 1). This suggests the fixation of mutations for MDR in the resistant isolates. Significant increase in the levels of erythromycin MIC was observed in the population of ciprofloxacin-exposed isolates compared to initial planktonic isolates and compared to antibiotic-free biofilms (Supplementary Fig. 1). Similar pleiotropic effects have been observed in studies with planktonic *Escherichia coli*,^27,28^ whereas our study demonstrates that these effects also extend to biofilm communities. However, for tetracycline-exposed isolates, no statistically significant net difference was observed overall between the erythromycin MIC levels of tetracycline-exposed isolates, antibiotic-free biofilms, and initial planktonic isolates (Supplementary Fig. 1), reinforcing the fact that tetracycline exposure had a limited effect on the development of cross-resistance in the population of antibiotic-exposed cells. No significant changes were observed in the levels of colistin resistance between the treatments, although sporadic emergence of resistance was observed.

Unexpectedly, more than two-thirds of cells recovered from antibiotic-free biofilms also developed a moderate increase in tetracycline resistance, and, overall, exhibited a statistically significant increase in net tetracycline resistance compared to planktonic isolates (*p* value = 0.013) (Fig. 2). Interestingly, as was observed in the biofilm formation assay (Fig. 1), tetracycline exposure also led to enhanced biofilm formation seen in many tetracycline-exposed isolates, and in a slight increase in the net biofilm formation among the 30 tetracycline-exposed isolates, compared to isolates from antibiotic-free biofilms (*p* value = 0.039). Such effects suggest a mild synergistic effect between mechanisms of biofilm formation and tetracycline resistance. It is widely accepted that enhanced biofilm formation leads to an increased resistance to various antibiotics and other environmental stressors via biofilm-specific resistance mechanisms.^5^ This indicates that tetracycline may act to promote MDR by enhancing the protection mechanisms provided by the biofilm. Such biofilm-specific effects are overlooked in broth microdilution MIC assays that test resistance levels in a planktonic state, and as a consequence, do not detect a significant net increase in antibiotic resistance in the MIC assay for tetracycline-exposed isolates (Fig. 2, Supplementary Fig. 1).

In contrast, ciprofloxacin exposure leads to the development of MDR via classical “cellular level” mechanisms^5^ that operate in individual cells. These effects result in significantly increased antibiotic resistance that can be detected by the broth microdilution MIC assays performed on planktonic cells (Fig. 2, Supplementary Fig. 1). Thus, our data suggest two distinct pathways for the development of MDR under ciprofloxacin *vs*. tetracycline exposure: (1) via increasing “cellular” drug resistance mechanisms (as tested in the MIC assay), especially seen for ciprofloxacin, often with a cross-resistance towards tetracycline and erythromycin, and (2) via increasing biofilm formation (as seen in the biofilm formation assay), as suggested for tetracycline.

### Genomic DNA sequencing and mutation analysis: Network analysis

Cells dispersing from biofilms have been reported to have high rates of phenotypic variation.^29^ To investigate the potential genetic basis of diversity in biofilm effluent cells, we sequenced the genomes of 30 random isolates from each treatment type, as well as the initial inoculum. Genome sequencing detected multiple mutations in each isolate (Supplementary Data 1). The majority of mutations were insertions and deletions and mutations mediated by the IS*Aba13* mobile element, followed by synonymous/intergenic and non-synonymous single-nucleotide polymorphisms (SNPs). A number of mutations mediated by mobile genetic elements IS*Aba1* and IS*Aba125* were also detected, as well as loss of plasmids 1 and 2 from various isolates (Table 1).

**Table 1:** Summary of mutations detected in P, B, BC, and BT samples, and the distribution of mutations across the three biological replicates (represented by R1, R2, and R3, respectively)

	P				B				BC				BT				
	R1	R2	R3	Total (P)	R1	R2	R3	Total (B)	R1	R2	R3	Total (BC)	R1	R2	R3	Total (BT)	Total (per mutation type)
SNP non-synonymous	1	1	1	3	4	2	9	15	9	6	3	18	4	1	9	14	50
SNP synonymous	4	2	1	7	9	4	4	17	12	6	7	25	7	9	10	26	75
SNP intergenic	10	0	2	12	8	0	6	14	10	0	1	11	17	0	1	18	55
Insertions/deletions	1	12	5	18	6	7	9	22	6	13	8	27	10	21	10	41	108
Loss of plasmid 1	1	3	6	10	5	7	6	18	3	4	1	8	1	1	1	3	39
Loss of plasmid 2	0	0	2	2	0	1	1	2	0	0	0	0	0	0	0	0	4
IS*Aba1* mediated	1	1	0	2	1	5	0	6	0	0	9	9	2	1	2	5	22
IS*Aba13* mediated	10	1	9	20	12	4	10	26	21	16	8	45	25	10	7	42	133
IS*Aba125* mediated	0	0	0	0	1	0	1	2	0	3	0	3	2	6	1	9	14
Total (per replicate)	28	20	26		46	30	46		61	48	37		68	49	41		
Total (per sample type)	74				122				146				158				

*P* planktonic samples, *B* antibiotic-free biofilm samples, *BC* ciprofloxacin-exposed biofilm samples, *BT* tetracycline-exposed biofilm samples

From planktonic growth experiments, the doubling time of AB5075 in a rich medium at 37 °C is estimated to be ~ 1 h,^30^ and the spontaneous mutation rate of multidrug resistant *A. baumannii* strains can vary from 0 to 2.1 × 10^−6^ mutations per cell division.^31^ However, the effects of doubling time and mutation rate on the number of mutations observed are not straightforward. The timing of mutation occurrence has a great impact on the overall number of mutations detected, since the earlier a mutation occurs, the greater the likelihood; it will be passed on and multiplied within subsequent generations, compared to a mutation appearing at a later timepoint. Furthermore, the biofilm mode of growth represents an additional challenge for such calculations, due to the heterogeneity and differences in the cellular activity across the biofilm layers.^32,33^ Therefore, we chose not to make such calculations and estimates, instead using de facto results on the number and nature of mutations as detected based on bioinformatic analyses.

Although it is hard to estimate exact mutation rates in biofilms, the number of mutations observed in this study was surprisingly high. A study by Hammerstrom et al.^34^ showed the emergence of a hypermutator phenotype characterized by inactivation of the *mutS* gene (encoding an essential protein for the DNA mismatch repair) in *A. baumannii*, when grown to evolve tigecycline resistance over 26 days, with gradually increasing concentrations of tigecycline, up to 32 times the initial MIC. No *mutS* gene mutations were observed in our data (Supplementary Data 1). The emergence of several mutations, mainly SNPs, and the development of resistance towards streptomycin was reported in a recent study of planktonically grown *Salmonella enterica* serovar Typhimurium LT2, in the presence of sub-inhibitory concentrations of streptomycin.^35^ Our study showed an abundance of not only SNPs but also a high number of mutations related to structural rearrangements such as insertions and deletions. The latter may not be unexpected as biofilms are known to promote genomic rearrangements and increased rates of horizontal gene transfer.^36^

Cells from antibiotic treatments displayed a higher number of IS-mediated mutations compared to antibiotic-free samples (Table 1), in agreement with reports that these mobile elements increase their rates of mobilization in the presence of antibiotics.^37^ Conversely, fewer instances of plasmid loss were detected in antibiotic-exposed cells, despite the fact that neither of the *A. baumannii* plasmids carry known ciprofloxacin or tetracycline resistance genes.

#### Description of main correlation patterns in network analysis

To examine the emergence of specific mutations in each sample type, as well as their possible effect on phenotype, network analyses were performed. These analyses were based on co-occurrence patterns (i) between the presence of specific mutations and the sample type/growth regime, (ii) between the presence of specific mutations and phenotypic traits (antibiotic resistance measured by the MIC analyses, and biofilm formation tested in the microtiter plate assay), and (iii) between specific mutations and biological replicates.

A strong positive correlation between a mutation and a sample type suggests that the particular treatment regime acted as a selective pressure that favored the fixation of that mutation, because the mutation conferred a phenotypic advantage under those growth conditions. Conversely, negative correlations indicate the reduced likelihood of certain mutations being fixed under particular growth conditions.

A positive correlation between a gene mutation and a phenotype implies a positive effect of that mutation on the emergence of the given phenotype, while negative correlations imply a negative effect on a given phenotype.

Positive/negative correlations between a mutation and a biological replicate indicate the prevalence/absence of that mutation in that biological replicate.

#### Correlations directly linking specific mutations with sample origin and/or a phenotype

**Fig. 3:**
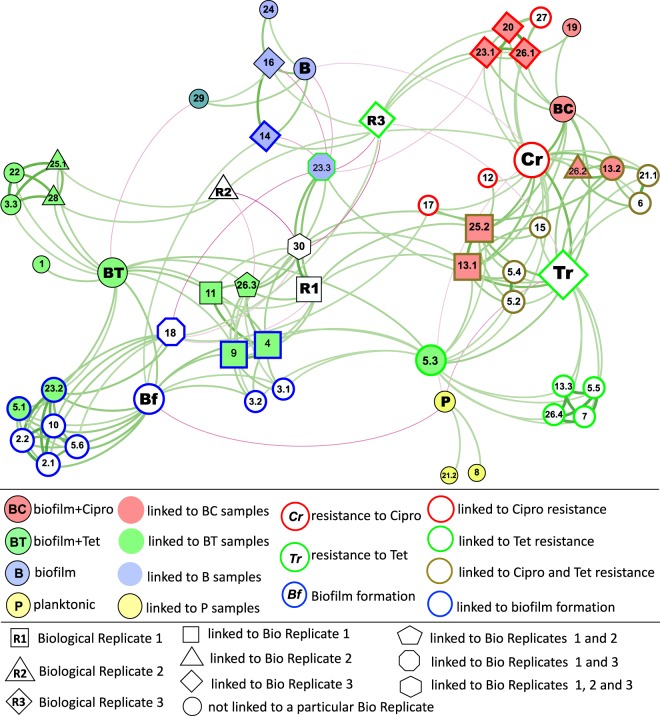
The network linking mutations with the growth regime, phenotypes, and biological replicates. The force-directed representation of the network is constructed based on co-occurrence patterns and correlations (*p* value <0.01) between mutations and the growth regime, between mutations and phenotypic measures, and between mutations and biological replicates. Growth regime/sample types are presented as color-filled nodes: P—planktonic culture (yellow-filled), B—antibiotic-free biofilm effluent (blue-filled), BC—ciprofloxacin-exposed biofilm effluent (red-filled), BT—tetracycline-exposed biofilm effluent (green-filled). Phenotypes are presented as color-outlined nodes: Bf (blue-outlined)—biofilm formation, measured in microtiter plate assay; Cr (red-outlined)—resistance to ciprofloxacin measured in MIC assay; and Tr (green-outlined)—resistance to tetracycline measured in MIC assay. P, B, BT, and BC nodes represent the sample type; Bf, Cr, and Tr nodes represent a phenotype; R1, R2, and R3 represent biological replicates 1, 2, and 3, respectively; all other nodes denote mutations directly linked with a specific sample type and/or phenotype. The fill color of nodes corresponds to the sample type that the mutation directly correlates with (linked to P—yellow-filled, to B—blue-filled, to BC—red-filled, to BT—green-filled). The node outline color corresponds to the phenotype with which the mutation is directly linked to (linked to Bf—blue-outlined, to Cr—red-outlined, to Tr –green-outlined). Mutations directly linked to both ciprofloxacin and tetracycline resistance are outlined in khaki. Mutations linked to a particular biological replicate are indicated as squares (linked to replicate 1), triangles (linked to replicate 2), diamonds (linked to replicate 3), pentagons (linked to replicates 1 and 2), octagons (linked to replicates 1 and 3), and a hexagon (linked to replicates 1, 2, and 3). The size of the node is relative to the node authority. Edges (the lines connecting the nodes) represent correlations between two nodes, positive correlations are presented in green, and negatives in magenta. Edge thickness/intensity represents the strength of correlation. The full description of each mutation is presented in Table 2

Several mutations in locus ABUW_0885 (mainly synonymous SNPs; nodes labeled 5.1–5.6 in Fig. 3) were linked with resistance to both antibiotics, as well as to enhanced biofilm formation. Despite a relatively large number of synonymous/intergenic SNPs included in the analyses (Table 1), only those five synonymous SNPs directly correlated with either a specific sample type or a phenotype, and, interestingly, all five synonymous mutations were in the same locus ABUW_0885 (nodes 5.2–5.6 in Fig. 3, Table 2). The limited effect of synonymous mutations may not be surprising as they do not change protein sequence, and, hence, often do not impact cellular fitness. However, in some cases synonymous SNPs can contribute to changes in phenotype and undergo selection.^38^ ABUW_0885 encodes a large, possibly secreted, protein often annotated as “biofilm-associated protein” (Bap). Bap proteins of *A. baumannii* are important for adherence to surfaces, including human epithelial cells, and for the development of the three-dimensional architecture of mature biofilms.^39^ Sequence analysis of the protein encoded by ABUW_0885 using the InterPro tool^40^ revealed multiple repeats, immunoglobulin (Ig)-like folds, and a type I secretion C-terminal target domain. The relatively high number of point mutations identified in ABUW_0885 suggests that it might be a hot spot for genetic and phenotypic variability. Similar repeat-containing genes with a high occurrence of mutations have been previously termed “contingency loci”—regions of hypermutable DNA that mediate high-frequency, stochastic, heritable changes that drive rapid evolution and adaptation of bacteria under changing environmental conditions.^41^

**Table 2:** Description of mutations that directly correlate (*p* value <0.01) with a growth regime, a phenotype, or a biological replicate

		Label	Mutation	Annotation	Gene/s	Gene product/s
SNPs	Non-synonymous	2.1	A → G	K5E (AAA → GAA)	*rplX*	50S ribosomal protein L24
		2.2	A → T	K6N (AAA → AAT)	*rplX*	50S ribosomal protein L24
		4	G → A	Intergenic (−98/−30)	ABUW_0747/ABUW_0748	Putative transcriptional regulator Cro/CI family/HP
		5.1	A → C	E2690A (GAG → GCG)	ABUW_0885	Biofilm-associated protein
		6	C → T	P233L (CCG → CTG)	ABUW_0944	Oxidoreductase α (molybdoprotein)-subunit
		10	C → T	A20V (GCA → GTA)	ABUW_1489	CsuB, putative secreted protein related to type I pili
		13.1	C → A	G318V (GGC → GTC)	*adeS*	AdeS kinase
		13.2	C → T	G318D (GGC → GAC)	*adeS*	AdeS kinase
		13.3	C → A	D167Y (GAT → TAT)	*adeS*	AdeS kinase
		14	G → A	R41H (CGT → CAT)	ABUW_2055	Fimbrial protein
		17	T → A	L102I (TTA → ATA)	ABUW_2540	Transposase
		27	C → T	Intergenic (+446/−139)	ABUW_4067/ABUW_4068	HP/HP
		30	G → A	Intergenic (+110/−135)	ABUW_1900/ABUW_1901	Oxidoreductase FAD, FMN binding/HP
	Synonymous	5.2	A → G	A2635A (GCA → GCG)	ABUW_0885	Biofilm-associated protein
		5.3	T → C	G1510G (GGT → GGC)	ABUW_0885	Biofilm-associated protein
		5.4	A → G	L2636L (TTA → TTG)	ABUW_0885	Biofilm-associated protein
		5.5	A → T	V2367V (GTA → GTT)	ABUW_0885	Biofilm-associated protein
		5.6	T → A	T1913T (ACT → ACA)	ABUW_0885	Biofilm-associated protein
InDels	Insertions	1	(CTTTGGATT)10 → 11	Intergenic (+257/−590)	*tyrS*/ABUW_0015	Tyrosyl-tRNA synthetase/16S rRNA gene
		8	+C	Intergenic (−206/−47)	ABUW_1059/ABUW_1060	HP/HP
		25.1	(T)5 → 6	Coding (737/1212 nt)	ABUW_3824	Family 1 glycosyl transferase
	Deletions	3.1	(A)5 → 4	Coding (236/1239 nt)	ABUW_0633	Putative methyltransferase
		15	(A)7 → 6	Coding (198/888 nt)	ABUW_2169	Putative membrane protein
		18	Δ1 bp	Coding (85/708 nt)	*vfr*	Virulence factor regulator, cAMP receptor-like protein
		19	Δ1 bp	Intergenic (−132/+517)	ABUW_3017/ggt	Integrase/gamma-glutamyltransferase
		21.1	(CGGTGCAGT)19 → 7	Coding (313–420/1368 nt)	*filE*	Pilus assembly protein
		21.2	Δ135 bp	Coding (421–555/1368 nt)	*filE*	Pilus assembly protein
		22	Δ141 bp	Partial loss of the gene	[ABUW_3448]	Glycosyl transferase, group 1
IS*Aba1*	Insertions	3.2	IS*Aba1* (–) + 9 bp	Coding (356–364/1239 nt)	ABUW_0633	Putative methyltransferase
		11	IS*Aba13* (–) + 9 bp	Coding (327–335/654 nt)	ABUW_1731	Transcriptional regulator, TetR family
		20	IS*Aba1* (+) + 9 bp	Coding (169–177/477 nt)	*smpB*	SsrA-binding protein
		26.1	IS*Aba1* (+) + 9 bp	Coding (229–237/1254 nt)	ABUW_3825	HP
IS*Aba13*	Insertions	16	IS*Aba13* (–) + 9 bp	Coding (170–178/1470 nt)	ABUW_2208	Adenylate guanylate cyclase
		23.1	IS*Aba13* (+) + 9 bp	Coding (70–78/327 nt)	ABUW_3609	DNA-binding protein HNS
		23.2	IS*Aba13* (–) + 9 bp	Coding (70–78/327 nt)	ABUW_3609	DNA-binding protein HNS
		23.3	IS*Aba13* (+) + 9 bp	Intergenic (−27/+160)	ABUW_3609/ABUW_3610	DNA-binding protein HNS/thioesterase superfamily protein
		25.2	IS*Aba13* (–) + 9 bp	Coding (779–787/1212 nt)	ABUW_3824	Family 1 glycosyl transferase
		26.2	IS*Aba13* (+) + 9 bp	Coding (1008–1016/1254 nt)	ABUW_3825	HP
		26.3	IS*Aba13* (+) + 9 bp	Coding (1224–1232/1254 nt)	ABUW_3825	HP
	Deletions	7	Δ9965 bp	IS*Aba13*-mediated	[ABUW_1758]–ABUW_1764	Putative acetyl esterase/lipase; extracellular serine protease; sulfate permease; HP; gdhB2 quinoprotein glucose dehydrogenase-B; UspA domain protein; GGDEF family protein
		9	Δ8706 bp	IS*Aba13*-mediated	ABUW_1759–ABUW_1764	Extracellular serine protease; sulfate permease; HP; gdhB2 quinoprotein glucose dehydrogenase-B; UspA domain protein; GGDEF family protein
		12	Δ871 bp	IS*Aba13*-mediated	[ABUW_1764]	GGDEF family protein
		24	Δ34,453 bp	IS*Aba13*-mediated	ABUW_3804–[ABUW_3830]	HP; HP; FE/S-dependent 2-methylisocitrate dehydratase; 2-methylcitrate synthase; methylisocitrate lyase; GntR family transcriptional regulator; aromatic amino acid aminotransferase; d-lactate dehydrogenase; K-capsule biosynthesis genes
IS*Aba125*		3.3	IS*Aba125* (+) + 3 bp	Coding (696–698/1239 nt)	ABUW_0633	Putative methyltransferase
		26.4	IS*Aba125* (+) + 3 bp	Coding (695–697/1254 nt)	ABUW_3825	HP
		28	Δ1985 bp	Between IS*Aba125*	ABUW_4087–ABUW_4089	HP; transposase
Loss of plasmid		29	Δ83,610 bp	Loss of plasmid 1	[repAci6]–ABUW_4123	Loss of plasmid 1

Labels correspond to node labels presented in Fig. 3. In the “Mutation” and “Annotation” columns, for SNPs the arrow shows changes in the codon. In the “Mutation” column, short repeat insertion/deletions are presented as the repeat sequence, in parentheses, followed by the number indicating changes in the number of corresponding repeat; for IS-mediated insertions—the IS element involved is given, followed by the strand (“+” or “−”), followed by the number of base pairs involved in the target site amplification. In “Annotation” column, for intergenic mutations, numbers within parentheses represent the position of the mutation, in nucleotide numbers, relative to the two neighboring genes: upstream (with “−” sign) or downstream (with “+” sign) of each gene. For InDels, the numbers within parentheses represent the positions of nucleotides affected in a coding sequence, out of the full number of the nucleotides in the gene. In the “Gene Product/s” column “HP” denotes a hypothetical protein. The slash separates the two genes on each side of an intergenic mutation

In ciprofloxacin-exposed samples, mutations were often directly linked to increased ciprofloxacin resistance, or resistance to both ciprofloxacin and tetracycline (as tested in MIC assays) (Fig. 3, Table 2). These include mutations in *smpB* and ABUW_3609 (nodes 20 and 23.1, respectively). SmpB in association with SsrA (tmRNA) plays an important role in rescuing stalled ribosomes and detoxifying toxic protein products under stress conditions. In earlier studies, deletion of these genes led to increased susceptibility to a range of antibiotics and environmental stresses,^42^ but increased resistance to fluoroquinolones, possibly due to a preventive effect on chromosome fragmentation.^43^ Likewise, in our study a mutation in *smpB* could have decreased or abolished the activity of the protein, leading to similar effects specific to the fluoroquinolone ciprofloxacin.

**Fig. 4:**
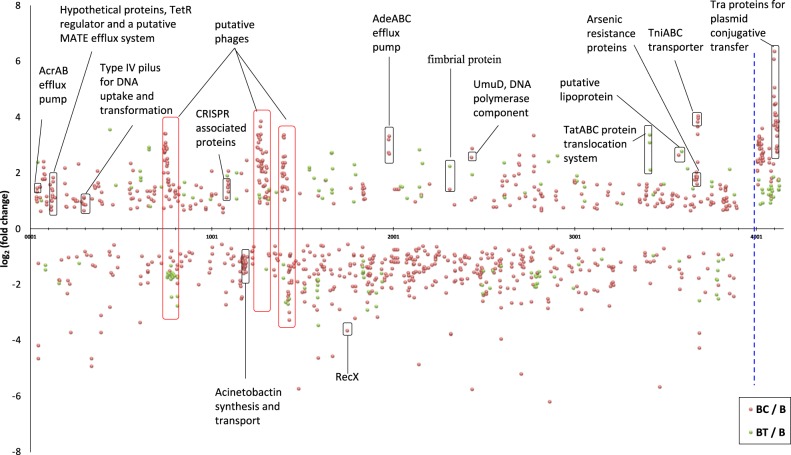
Transcriptomic changes observed in antibiotic-exposed biofilms compared to antibiotic-free biofilm samples. Transcriptomic changes (log_2_ fold change; *p* adj <0.05) detected in ciprofloxacin- and tetracycline-exposed biofilm samples are presented by orange and green circles, respectively. The *X*-axis represents locus tag numbers of the AB5075-UW main chromosome and the largest plasmid 1 (separated by the blue dashed line). Putative phage genes are outlined by red rectangles. Three independent biological replicates were used for evaluating significance

Three types of non-synonymous point mutations were detected in the *adeS* gene in effluent cells from ciprofloxacin-exposed biofilms (nodes 13.1–13.3 in Fig. 3, Table 2). Two of these affected codon 318 and resulted in the substitution of glycine with valine or aspartic acid, respectively (nodes 13.1 and 13.2 in Fig. 3, Table 2). These mutations showed strong association with resistance to both tetracycline and ciprofloxacin in the network analysis. The third non-synonymous point mutation (node 13.3), affecting codon 167, showed a weak association with tetracycline resistance (Fig. 3). AdeS is a sensor which, in conjugation with the AdeR response regulator, regulates the expression of the AdeABC RND family multidrug efflux system—one of the major mechanisms of MDR in *A. baumannii*. Mutations in *adeS* can lead to the constitutive expression or overexpression of this efflux system.^44,45^ Corroborating our mutation analysis, the AdeABC efflux pump was highly up-regulated in the transcriptomic data from ciprofloxacin-exposed biofilm samples, and, to a lesser degree, in tetracycline-exposed biofilm samples and antibiotic-free biofilms (Fig. 4, Supplementary Fig. 2, Supplementary Data 3). Based on InterPro analysis,^40^ codon 318 is located within the ATPase domain of the *adeS* histidine kinase, whereas codon 167 is within the dimerization/phospho-acceptor domain. Thus, in addition to identifying gene mutations linked to particular phenotypes, our data can identify mutations in different parts of a gene that may have different phenotypic impacts. In the case of *adeS* histidine kinase, mutations in the ATPase domain, particularly those affecting codon 318, may have greater impact on the expression of the *adeS-*regulated AdeABC efflux pump, and, subsequently, on antibiotic resistance, compared to mutations in phospho-acceptor domain.

Mutations in two neighboring genes, ABUW_3824 (nodes 25.1 and 25.2 in Fig. 3, Table 2) and ABUW_3825 (nodes 26.1–26.4), encoding a family 1 glycosyl transferase and a hypothetical protein, respectively, were common in both ciprofloxacin- and tetracycline-exposed biofilm samples. These mutations were often linked with antibiotic resistance phenotypes, suggesting that they might confer antibiotic resistance and were selected in the presence of antibiotics (Fig. 3). Genomic analysis showed that these genes are within the K-locus, which determines the production of capsular polysaccharide known to protect against killing by host serum, and to increase virulence.^46,47^ The K-locus in strain AB5075-UW includes genes with locus tags ABUW_3815–ABUW_3833, flanked by *lldP* and *fkpA*. A similar organization of the K-cluster occurs in other *A. baumannii* strains.^46^ Geisinger and Isberg^47^ have reported an increase in the K-locus exopolysaccharide when grown in the presence of sub-inhibitory concentrations of chloramphenicol and erythromycin, due to increased transcription of K-locus genes.^47^ Whether mutations in K-locus genes identified in our study lead to changes in the production in the capsular polysaccharides is not known. However, our study demonstrates mutational changes in the K-locus at a genomic level in the biofilm effluent cells with the potential to translate into fixed alterations in the production of the polysaccharide. In our transcriptomic data, various sets of K-locus genes were significantly up- or down-regulated depending on the growth regime (Supplementary Fig. 2, Supplementary Data 3), suggesting that the treatments might result in variation of the K-capsule structure.

IS*Aba13* mobile element-mediated mutations affecting ABUW_3609 were detected in antibiotic-exposed biofilm samples (nodes 23.1–23.3 in Fig. 3, Table 2). This locus encodes the DNA-binding H-NS protein. These mutations were linked to resistance phenotypes, as well as to enhanced biofilm formation (Fig. 3). IS-mediated mutations in an H-NS protein have been linked to high-level colistin resistance in *A. baumannii*^48^ and led to enhanced adherence to human pneumocytes and an increase in virulence.^49^ The latter was accompanied by up-regulation of type VI secretion system and pili. Up-regulation of these genes was also observed in our transcriptomic data (Supplementary Fig. 2, Supplementary Data 3). Taken together, these suggest that antibiotic exposure may facilitate the emergence of H-NS mutants, and, subsequently, lead to increased antibiotic resistance and virulence. H-NS has several roles in the cell, most notably in gene regulation and the silencing of horizontally acquired foreign DNA that often encodes virulence factors and antibiotic resistance determinants.^50^ The mutations affecting ABUW_3609 may lead to inactivation of this gene, and, therefore, activation or “de-silencing” of horizontally acquired genes. These may include genes involved in antibiotic resistance, resulting in the emergence of antibiotic-resistant phenotypes.

Mutations in ABUW_2055, encoding a fimbrial adhesin, appeared in antibiotic-free biofilm samples and had a positive correlation with increased biofilm formation (node 14 in Fig. 3). The link between ABUW_2055 and biofilm formation is not surprising as fimbrial adhesins, also called attachment pili, are polymeric fibers that play an important role in surface attachment and biofilm formation.^51^

A mutation in *csuB* (ABUW_1489), part of the *csu* operon that codes for proteins involved in a chaperon-usher pili assembly system important for pilus assembly and biofilm formation,^52,53^ was linked to increased biofilm formation in our analyses (node 10 in Fig. 3). Peleg et al.^54^ showed that the *csu* operon was only present in pathogenic strains of *A. baumannii*, suggesting this is an important virulence factor.

A mutation in *vfr* (node 18 in Fig. 3) encoding the virulence factor regulator was also positively correlated with biofilm formation. Up-regulation of *vfr* was observed in the transcriptomic data of biofilm samples (Supplementary Data 3). This locus has been implicated in quorum sensing and flagellar biogenesis,^55,56^ both of which are important determinants of biofilm formation.

#### Correlations between tetracycline exposure and biofilm formation

Several genes were commonly mutated in tetracycline-exposed samples. However, these mutations often positively correlated with increased biofilm formation rather than increased resistance to tetracycline, as can also be seen by the close proximity of nodes representing tetracycline exposure and biofilm formation (nodes BT and Bf, respectively, Fig. 3). This reinforces a possible synergy between biofilm formation and tetracycline resistance mechanisms. Among these mutations was a large 8706 bp deletion involving genes ABUW_1759–ABUW_1764 found in tetracycline-exposed cells and linked to enhanced biofilm formation (node 9, Fig. 3). This region encodes several proteins, including a diguanylate cyclase/phosphodiesterase containing GGDEF and EAL domains. This protein is involved in the regulation of c-di-GMP levels and is known to affect physiological processes including biofilm formation.^57^

Mutations in a putative SAM-dependent methyltransferase (ABUW_0633) also arose predominantly in tetracycline-exposed biofilm dispersal cells (Fig. 3). Mutations in SAM-dependent methyltransferases have been linked to the increased resistance to doxycycline and tigecycline.^58,59^ Considering the structural similarities of these two antibiotics with tetracycline (all belonging to the tetracycline class of antibiotics), we can speculate that mutations in ABUW_0633 also have a role in increasing tetracycline resistance. Tetracycline-class antibiotics inhibit protein synthesis by preventing the aminoacyl tRNA subunit from binding to the acceptor site of the 30S ribosomal subunit. Webb et al.^58^ suggested that a SAM-dependent methyltransferase that has been shown to confer resistance to doxycycline in in *Burkholderia pseudomallei* probably methylates the tetracycline binding site of the 30S ribosomal subunit. Subsequently, alteration of this gene function through mutation or deletion might change ribosomal methylation patterns, which in turn decreases binding efficiency.^58^ However, based on our analysis, none of the mutations in ABUW_0633 (nodes 3.1–3.3 in Fig. 3) was directly linked with tetracycline resistance, but mainly correlated with increased biofilm formation. This suggests that increased biofilm formation in these cells may be at least partially responsible for the increased tetracycline resistance.

The intergenic mutation between ABUW_0747 and ABUW_0748 that can affect the promoter region of the two genes (node 4 in Fig. 3) predominantly occurred in tetracycline-exposed isolates. The presence of this mutation also correlated with biofilm formation and not tetracycline resistance. ABUW_0747 and ABUW_0748 are part of a prophage (predicted to include genes ABUW_0733–ABUW_0815^60^) and encode Cro/CI family transcriptional regulators involved in controlling the life cycle of the phage and its transformation into the lytic phase. Clusters of genes in this phage were significantly up and down-regulated during biofilm growth in our transcriptomics data, and were mainly down-regulated under tetracycline exposure (Fig. 4, Supplementary Fig. 2, Supplementary Data 3). Bacteriophage life cycles have been linked with biofilm development.^61^ Whether the putative phage ABUW_0733–ABUW_0815 has a role in biofilm formation in *A. baumannii* and/or in antibiotic resistance is not known.

Thus, our study revealed an interesting phenomenon. Mutations that appeared in the tetracycline-exposed cells were primarily linked with increased biofilm formation, and not directly with increased tetracycline resistance, based on the *p* value threshold for each correlation. Corroborating these data, the biofilm formation assays showed that many tetracycline-exposed isolates had a notable increase in their capacity to form biofilms, compared to antibiotic-free biofilm effluent isolates (Fig. 1), suggesting that tetracycline exposure may drive the emergence of mutations that result in increased biofilm formation. Ultimately, such events could lead to serious consequences, as increased biofilm formation can result in increased resistance to a wide range of antibiotics and environmental stressors via biofilm-specific resistance mechanisms.^5^

#### Loss of plasmid 1

Complete loss of the largest plasmid, plasmid 1 (shown as node 29 on Fig. 3) positively correlated with antibiotic-free biofilm samples, indicating this event was common in antibiotic-free biofilms. However, loss of plasmid 1 was negatively correlated with tetracycline exposure, suggesting there may be selective pressure to retain plasmid 1 in the presence of tetracycline. Interestingly, despite having several annotated genes involved in resistance towards antibiotics such as streptomycin, no tetracycline resistance genes are known to be harbored on plasmid 1. Similarly, no loss of plasmid 2 was observed for antibiotic-exposed cells (Table 1).

#### Differences in mutational spectra between biological replicates

As demonstrated in Fig. 3, there were only a limited number of mutations that were prevalent or absent in specific biological replicates, as evident by few positive and negative edges linking a particular replicate (represented by nodes R1, R2, and R3) with specific mutations. Moreover, the nodes representing biological replicates are relatively centrally located within the network, in close proximity to each other. This implies that there were strong similarities between biological replicates in their overall mutational spectrum, and none of the replicates showed a strong inclination towards a specific growth regime or a phenotype. In accordance with the latter, the nodes representing biological replicates had no direct correlation with a growth regime or a phenotype, except biological replicate 3 (node R3) that showed a negative correlation with tetracycline resistance (node Tr). Corroborating this observation from the network analysis, in our MIC assay the isolates originating from biological replicate 3 showed notably decreased tetracycline resistance compared to isolates from other replicates (Fig. 2).

All three biological replicates correlated with an intergenic SNP indicated by node 30, replicate 1 showing a strong positive correlation, while replicates 2 and 3 displayed negative correlations with this node in the network figure (Fig. 3). Apart from these correlations, the mutation represented by node 30 was not directly linked with a particular phenotype or a sample type, and, thus, had no phenotypic implications and was not enriched in a particular sample type. Upon inspection (Supplementary Data 1), it became apparent that this mutation was consistently and exclusively detected in isolates carrying numbers 101–110, from all sample types P, B, BC, and BT. This implies that this mutation was present in the original clone used to inoculate the overnight culture (from which the planktonic isolates P101–P110 were obtained) for biological replicate 1 and was also detected in nearly all random biofilm isolates originating from biological replicate 1. The mutation was absent in isolates originating from replicates 2 and 3. This shows that there was a degree of variability among the initial clones used for inoculation of triplicate overnight cultures and, once again, highlights the power of our mutation analysis.

#### Correlations between mutations

Multiple correlations were observed between mutations, highlighting the potential for interplay between genes in generating advantageous phenotypes (Fig. 3, Table 2, Supplementary Data 2).

A strong positive correlation between two mutations indicates that these mutations often co-occur within the same genome, for which there are two likely explanations: there are (i) cumulative effects where the two mutations may be co-dependent and act in tandem, possibly having a synergistic effect on the phenotype; or (ii) compensatory effects where emergence of one mutation under given growth conditions requires a second mutation to rescue any fitness defect imposed by the first mutation.

There were strong positive correlations observed between the IS*Aba1*-mediated mutation in the *smpB* gene (node 20 in Fig. 3, Table 2) and the insertion of IS*Aba13* into ABUW_3609, encoding a DNA-binding H-NS protein (node 23.1). These mutations emerged together in ciprofloxacin-exposed samples, were directly linked with ciprofloxacin resistance, and, according to the literature, might play a role in chromosome architecture and stability.^43,62^

Strong correlations were observed between all three mutations in the *adeS* gene (nodes 13.1–13.3 in Fig. 3), the sensor kinase involved in AdeABC efflux pump regulation, and mutations in either ABUW_3824 or ABUW_3825 (nodes 25.2, 26.2, and 26.4), involved in the biosynthesis of the K-capsule. This suggests a possible functional link between the two mechanisms. Both of these may affect membrane permeability of the antibiotics, and/or changes in the K-capsule might ameliorate toxic effects of AdeS-mediated overexpression of AdeABC. Such interconnectivity between gene functions remains largely unexplored.

Two mutations in *rplX* gene, encoding ribosomal protein L24, were linked with enhanced biofilm formation. These two mutations, leading to amino acid substitutions in positions 5 and 6 (nodes 2.1 and 2.2 in Fig. 3), were strongly correlated with each other and were only found as co-occurring mutations.

Also strongly linked were mutations in ABUW_3824 and ABUW_3448 (nodes 25.1 and 22 in Fig. 3), both of which encode similar glycosyl transferases. These two mutations in turn were also correlated with a mutation in ABUW_0633 (node 3.3) and the deletion of uncharacterized plasmid genes ABUW_4087–ABUW_4089 (node 28).

Negative correlations between mutations indicate that the two mutations are not likely to be present in the same genome, possibly due to the lethal, or highly disadvantageous phenotypic effects that one mutation may have in the presence of the other. Such relationships also suggest functional links between affected genes. An example is the intergenic mutation between the genes ABUW_3609–ABUW_3610 (node 23.3), which is negatively correlated with mutations in *vfr* (node 18), ABUW_2055 (node 14), and ABUW_2208 (node 16), many of which are enriched in the antibiotic-free biofilms or linked with increased biofilm formation (Fig. 3). It is likely that the intergenic mutation affects expression of ABUW_3609, as an IS*Aba13* insertion in ABUW_3609 (node 23.2) is linked with increased biofilm formation.

Interactions between various mutations observed in this study were unexpected, as these links had not been identified previously. Because our study involved whole genome-wide analysis of all mutations, it was possible to reveal multiple mutations per genome and, subsequently, investigate correlations between the presence and absence of mutations in different parts of the genome. Such analyses can identify novel interactions between genes, which have been previously overlooked, and provide targets for future studies.

### RNA-sequencing transcriptomics

As soon as effluent isolates were collected from each biofilm sample, whole biofilms were harvested and used for RNA extraction and transcriptomics to identify genes whose transcription was up- or down-regulated in each of the treatments. Nearly half the genes (1516 out of 3895 genes, including plasmid genes) of *A. baumannii* AB5075-UW were significantly (*p* adj. <0.05) up- or down-regulated in biofilms compared to stationary phase planktonic cultures (Supplementary Fig. 2, Supplementary Data 3). This reflects profound physiological differences between planktonic and biofilm lifestyles.

Among the most up-regulated genes in biofilms were genes involved in the synthesis of ribosomal proteins (Supplementary Fig. 2). This has been observed previously, for example, in comparisons between gene expression levels in biofilms and stationary phase cultures of *E. coli.*^63^ In *Gardnerella vaginalis*, ribosomal proteins were down-regulated in comparison to planktonic cells in exponential phase.^64^ Consequently, differences in the expression of ribosomal proteins are probably related to differences in cellular activity between the stationary phase cultures and exponentially growing planktonic cultures, whereby the ribosomal turnover in biofilms is higher compared to stationary phase planktonic cultures, but lower in comparison to exponentially growing planktonic cultures. A number of other genes involved in basic metabolic processes such as protein synthesis, carbohydrate metabolism, and cell division were also up-regulated in biofilms (Supplementary Fig. 2, Supplementary Data 3), further suggesting that differences in cellular activity lie behind the differential expression of genes involved in these processes.

In comparison to stationary phase planktonic cultures, genes encoding universal stress proteins and catalases were down-regulated in biofilms, supporting ideas that the stationary phase is characterized by nutrient limitation, changes in pH, and the accumulation of toxic by-products.^65,66^

Among the highly up-regulated and down-regulated genes in all biofilm samples were genes/gene clusters involved in the biosynthesis of proteins for type VI secretion systems, efflux, cell surface modification, and pili, as well as membrane proteins and lipoproteins of unknown function (Supplementary Fig. 2, Supplementary Data 3). These gene products often have important roles in cell interaction and communication, which is of paramount importance in biofilm formation and functioning. All biofilm samples, with or without antibiotic exposure, exhibited up-regulation of drug efflux transport systems. While these systems are best known for their role in MDR, they were up-regulated in biofilms even in the absence of antibiotics. This suggests that such systems have a biofilm-specific role that has been largely overlooked. There are a few reports that suggest a link between efflux transporters and biofilm formation. For example, an efflux transporter component TolC was found to promote cell aggregation in *E. coli.*^67^ In *P. aeruginosa*, an ABC-family efflux pump was preferentially expressed in biofilms compared to planktonic cells, conferring biofilm-specific antibiotic resistance.^68^ Despite these reports suggesting the importance of efflux in biofilms, the role of efflux transporters in biofilms remains largely unknown^69^

The operon that includes genes *nuoA-nuoN* (ABUW_3165–ABUW_3177) and encodes NADH dehydrogenase I was significantly up-regulated in biofilm samples (Supplementary Fig. 2). In the rhizosphere-dwelling bacterium *Pseudomonas fluorescens*, the *nuo*-encoded NADH dehydrogenase I was essential for plant root colonization.^70^ Since biofilm formation is an essential trait for colonization, we speculate that the *nuo* operon has a role in biofilm formation in *A. baumannii*.

To identify specific transcriptional responses to each antibiotic, transcriptomic data from antibiotic-exposed biofilm samples were compared to data from antibiotic-free biofilms (Fig. 3). The addition of antibiotics further increased the up-regulation of genes related to antibiotic resistance, most notably, the RND family efflux transport system AdeABC, involved in resistance to a variety of antibiotics.^71^ Several other putative multidrug efflux systems were also up-regulated. Ciprofloxacin exposure led to an increase in the expression of genes in phage and phage-like islands previously identified in the genome of AB5075-UW,^60^ whereas tetracycline generally had the opposite effect by down-regulating these genes (Fig. 4, Supplementary Fig. 2).

Ciprofloxacin exposure resulted in increased expression of *tniABC* genes involved in transposition, *tra* genes involved in translocation/plasmid conjugation, and the genes *recA* and *umuD* (Fig. 4, Supplementary Fig. 2). The DNA recombination and repair protein RecA, in conjunction with UmuD polymerase, plays a central role in the induction of SOS pathway of DNA repair and mutagenesis. The SOS response also promotes homologous recombination and horizontal gene transfer,^72^ processes important for phage transduction, conjugation, and DNA repair. The *recX* gene, which encodes the inhibitor of RecA and has a preventive effect on the induction of the SOS response,^73^ was significantly down-regulated under ciprofloxacin exposure.

Induction of *recA*-facilitated homologous recombination by ciprofloxacin has been previously reported in other bacteria.^74–76^ Up-regulation of phage transduction, type IV pili-related transformation systems, and plasmid conjugation genes observed in this study is probably the result of *recA-*mediated induction of the SOS pathway under ciprofloxacin exposure. Other genes up-regulated by ciprofloxacin included the CAS genes, part of the CRISPR-related defense system against bacteriophage and conjugative plasmid transfer. This is probably a consequence of the up-regulation of chromosomally encoded phage and plasmid-encoded conjugation genes.

Tetracycline exposure led to differential expression of uncharacterized hypothetical proteins, putative transporters, and the twin-arginine translocation *tatABC* genes involved in proofreading and translocation of large folded proteins across the cytoplasmic membrane^77^ (Fig. 4, Supplementary Fig. 2). High-level up-regulation of *tatABC* genes (up to 40-fold) suggests a possible novel mechanism of action for TatABC in tetracycline resistance in biofilms.

Intriguingly, among the most up- or down-regulated genes in biofilm samples were a large number of genes/gene clusters encoding hypothetical proteins with no known function (Supplementary Fig. 2, Supplementary Data 3). Differential expression in these genes reached more than 1000-fold highlighting significant gaps in our knowledge of how biofilms form and persist. These currently unknown gene products could be key factors in biofilm function and could be explored as targets for controlling biofilm formation in medical and environmental contexts.

### Implications and concluding remarks

Biofilms are recognized as the predominant lifestyle for the majority of microorganisms. They represent cell communities with significant physiological differences from their planktonic counterparts.^15,78,79^ Consequently, many processes that have been studied using planktonic cultures may not apply to biofilms. This is a particular problem for antibiotic resistance research, with the realization that microbial resistance in biofilms far exceeds the resistance levels observed in planktonic cultures.^13,21^ The unique properties of biofilms provide protection against antibiotics.^5^ Biofilm-specific processes such as cell differentiation and increased rates of horizontal gene transfer can further facilitate the dissemination of antibiotic resistance.^36^

Dispersal of cells from biofilms is an essential process in the biofilm life cycle and is associated with formation of genetic variants that help to ensure successful re-colonization,^80^ and genetic diversification during biofilm growth has been demonstrated in *P. aeruginosa.*^81^ Here, we demonstrate that the emergence of genomic variants within biofilm dispersal cells is largely synchronized with changes in the environment. Thus, after 6 days of biofilm growth, only 3 days of which were in the presence of sub-inhibitory concentration of antibiotics, *A. baumannii* biofilm dispersal cells had accumulated a wide diversity of mutations that conferred phenotypic changes that were advantageous in the presence of antibiotics. Moreover, we could also trace mutations to changes in specific gene expression profiles in these biofilms.

Our data demonstrate the remarkable ability of microorganisms to adapt to particular environments via rapid evolution, driven by generation of mutations that are subsequently fixed in cell populations. These genomic changes conferred fitness advantages that could overcome environmental pressures such as antibiotic exposure. In particular, our work demonstrates the alarming emergence of resistant phenotypes within a very short time period after exposure to sub-inhibitory concentrations of antibiotics. It provides us with an unprecedented window into the consequences of antibiotic exposure on bacteria in various natural and medical settings. These rapid and dramatic effects of antibiotic exposure on biofilm cells might not have been fully appreciated.

Sequencing technologies have developed to the point where whole genome sequencing has become a trivial task. Using DNA-sequencing to understand mutation, transcription, and phenotypic changes to organisms is now practical. We can move beyond understanding the effect of specific single mutations on phenotype. Direct investigation of the natural emergence of mutations under selective pressures via high-throughput genome sequencing, with minimal sample manipulation, allows an unbiased and potentially global understanding of bacterial evolutionary processes. The consequence of interplay between specific mutations can thus be studied.

In summary, our data highlight the genomic flexibility of bacteria to quickly adapt to changing environments. These responses can involve multiple mutations within a single genome that, combined, may generate novel phenotypes, thus challenging the “one mutation–one phenotype” paradigm. By using the power of large-scale genome sequencing and transcriptomics, it is possible to decipher processes and natural adaptations occurring in bacteria. This will help to identify mutations with potential roles in biofilm formation and antibiotic resistance. Since biofilms are the prevalent form of microbial life in many environments, the results of our study and our approach are applicable to a wide range of microorganisms, both in clinical and environmental settings. In turn, these findings will become a roadmap for future studies to focus on the role of newly discovered genes and gene combinations in the development of resistant phenotypes. These are potential targets in the war on bacterial resistance.

## Methods

### Strains and growth conditions

A previously sequenced and characterized, highly virulent strain of *A. baumannii*, AB5075-UW,^60,82^ was used. The strain was maintained on a cation-adjusted Mueller–Hinton (MH) medium (Becton, Dickinson and Company).

### Biofilm growth and antibiotic exposure

Biofilms were grown in Tygon R-3603 tubes (VWR International) attached to a peristaltic pump delivering fresh medium at a rate of 4 ml/h. Three single colonies of AB5075-UW were inoculated into liquid MH medium in three separate tubes (a separate colony per tube). One hundred microliters of diluted overnight culture of AB5075-UW (at 1.6 × 10^4^ cells per ml) were inoculated into sterile tubes, using three independent biological replicates for each treatment, each replicate in a separate tube/channel (three channels per treatment). After 3 days of growth at 37 °C to allow biofilm establishment, one set of triplicate samples was left antibiotic-free, while the MH medium for the second set was supplemented with 62.5 μg/ml ciprofloxacin (0.5 MIC), and the growth medium for the third set of samples was supplemented with 2 μg/ml tetracycline (0.25 MIC). In order to choose an appropriate concentration of antibiotics for these biofilm experiments, an MIC test was performed using the broth microdilution method as described below. The concentration of antibiotics chosen represented the minimum concentrations that excluded any growth inhibitory effects in the MIC test. After the addition of antibiotics, biofilms were grown for a further 3 days, at which time the biofilm effluent cells were collected for DNA-sequencing. Biofilms formed inside the tubes were harvested for use in RNA extraction and sequencing.

### DNA extraction, sequencing, and mutation analysis

After 72 h of growth, 1 ml of biofilm effluent containing cells detaching from biofilms were collected from each channel. Effluents were diluted 1000-fold and spread on fresh solid MH medium to grow colonies overnight. Ten random, distantly located single colonies were picked from each biological replicate, totaling 90 isolates. These included 30 biofilm effluent isolates grown under ciprofloxacin exposure (BC samples), 30 effluent isolates grown under the tetracycline exposure (BT samples), and 30 effluent isolates derived from antibiotic-free biofilms (B samples). Isolates were also collected from the initial overnight planktonic cultures that were used to inoculate biofilms by spreading the aliquot of the diluted culture, in corresponding biological triplicates, on MH solid medium and collecting 10 distantly located random single colonies per biological replicate, totaling 30 isolates derived from the initial planktonic culture (P samples).

Genomic DNA was extracted from all 120 isolates individually using the DNeasy kit (Qiagen) and the manufacturer’s protocol. Genomic DNA samples were submitted to the Ramaciotti Center for Gene Function Analysis (UNSW, Sydney, Australia), where samples were sequenced using HiSeq2500, yielding 120 million paired end reads of 250 bp in length.

Sequence data were trimmed from low-quality data using Trimmomatic software^83^ and the quality of sequence data was assessed using FastQC (Babraham Bioinformatics). Paired end reads were combined using Flash.^84^ The Breseq algorithm^85^ was used for mutation analysis of each individual sample, using combined sequencing reads derived from each isolate. The complete genomic sequence of AB5075-UW available from GenBank (Accession: PRJNA243297) was used as a reference. Mutations with <50% frequency (as estimated by Breseq) were excluded.

### Network analysis using mutation data

Pearson’s correlations based on mutation co-occurrences and quantitative phenotypic data were calculated in R using the Hmisc 4.1-0 package.^86^ The correlation data obtained (as presented in Supplementary Data 2) was imported into the network visualization software Gephi^87^ to generate networks based on the ForceAtlas2 layout algorithm.^88^ Only correlations with *p* value <0.01 were included in network analyses. Only mutations directly linked with major nodes (those representing growth regimes, phenotypes, and biological replicates) are shown. Full unfiltered correlation results are presented in Supplementary Data 2.

### RNA extraction, sequencing, and analysis

After 72 h of growth following the addition of antibiotics and immediately upon collection of biofilm effluent cells, tubes containing biofilms (three independent biological replicates for each treatment, each replicate in a separate tube/channel, as stated previously) were washed with MH medium to remove planktonic or loosely attached cells. Qiazol reagent was added directly to the biofilms in tubes to lyse and collect the cell material. RNA extraction was performed using the miRNeasy kit (Qiagen) according to the manufacturer’s protocol. RNA samples were submitted to the Ramaciotti Center for Gene Function Analysis (UNSW, Sydney, Australia) for ribosomal RNA depletion and sequencing. RNA samples were sequenced on the NextSeq500 platform generating 400 million paired end reads, 75 bp in length.

Sequence data were trimmed from low-quality data using Trimmomatic software.^83^ The quality of sequence data was assessed using FastQC (Babraham Bioinformatics). Genome mapping was performed using the EDGE-pro algorithm.^89^ Differential expression was calculated in R using the Deseq2 package.^90^ To validate the differential expression data, the RNA-sequencing analysis was also performed using the Rockhopper software^91^ and the Tophat/Cufflinks/Cuffdiff pipeline,^92^ both of which yielded results similar to the EDGE-pro/Deseq2 output.

### Quantification of biofilm formation

The capacity of each isolate to form biofilms was tested by the ability of the cells to adhere to the wells of 96-well microtiter dishes (Cellstar, flat bottom, Greiner Bio-One) followed by CV staining of the biofilms formed, as described by O’Toole and Kolter^93^ with slight modifications. Briefly, overnight cultures of each isolate, in triplicate, were inoculated (resulting in 1:100 dilution of the overnight culture) into wells of 96-well microtiter plates containing 100 μl cation-adjusted MH liquid medium. Inoculated plates were incubated at 37 °C with shaking (100 r.p.m.) for 20 h. After incubation, the growth was confirmed as the OD_600_ using a PHERAstar microplate reader (BMG Labtech), after which the liquid medium was removed from each well and the wells rinsed twice with phosphate-buffered saline (PBS, pH 7.4, Sigma) to remove loosely attached cells. A 0.2% (w/v) aqueous solution of CV stain (Sigma) was applied for 15 min followed by PBS washing, twice, to remove the excess stain. Ethanol (150 μl, 100%) was added to each well to extract the CV stain. The extract was diluted 5-fold and absorbance of the CV stain read at 590 nm using the microplate reader.

To evaluate net differences in biofilm formation between isolates originating from planktonic (P), antibiotic-free biofilm (B), ciprofloxacin-exposed biofilm (BC), and tetracycline-exposed biofilm (BT) samples, a three-level mixed-factor nested analysis of variance (ANOVA) was used (technical replicates nested within each isolate, isolates nested within samples in separate biological replicates, and biological replicates nested within sample types) revealing significant heterogeneity between samples (*F*_3.8_ = 12.97; *p* = 0.002). The post hoc Tukey’s HSD (honestly significant difference) test was employed to reveal differences between sample pairs.

### Antibiotic susceptibility testing

Antibiotic susceptibility of each isolate was tested using an MIC broth microdilution method,^94^ using 2-fold dilutions of antibiotics starting from 1000 μg/ml for ciprofloxacin, 125 μg/ml for tetracycline, 30 μg/ml for colistin, and 1000 μg/ml for erythromycin.

To evaluate net differences in MICs between isolates originating from P, antibiotic-free B, ciprofloxacin-exposed BC, and tetracycline-exposed BT samples, a mixed-factor nested ANOVA was used (MICs for individual isolates nested within samples in separate biological replicates, and biological replicates nested within sample types), followed by the post hoc Tukey’s HSD to reveal differences between sample pairs. The ANOVA test revealed statistically significant heterogeneity between samples in all MIC tests, including for ciprofloxacin (*F*_3,8_ = 40.83; *p* = 3.38E − 05), tetracycline (*F*_3,8_ = 5.22; *p* = 0.027), and erythromycin (*F*_3,8_ = 4.96; *p* = 0.031). Due to the nature of MIC microdilution assay, MIC values often do not follow a normal distribution, as was also apparent in our study when tested using data distribution histograms. To achieve normal distribution, the data was log-transformed prior to ANOVA calculations, as described in ref. .^95^

### Reporting summary

Further information on experimental design is available in the Nature Research Reporting Summary linked to this article.

## Supplementary information

Supplementary Information

Dataset 1

Dataset 2

Dataset 3

Reporting summary

## Data Availability

The DNA and RNA sequence data that support the findings of this study have been deposited in the NCBI Sequence Read Archive (SRA) with the accession number SRP155796.
