# Supplementary material for: Rapid microevolution of biofilm cells in response to antibiotics
Source: NPJ Biofilms Microbiomes. 2019 Nov 6;5:34. doi: 10.1038/s41522-019-0108-3 (PMC6834608; doi:10.1038/s41522-019-0108-3)
Supplement: Supplementary file 1 — Supplementary Information [file 41522_2019_108_MOESM1_ESM.pdf]

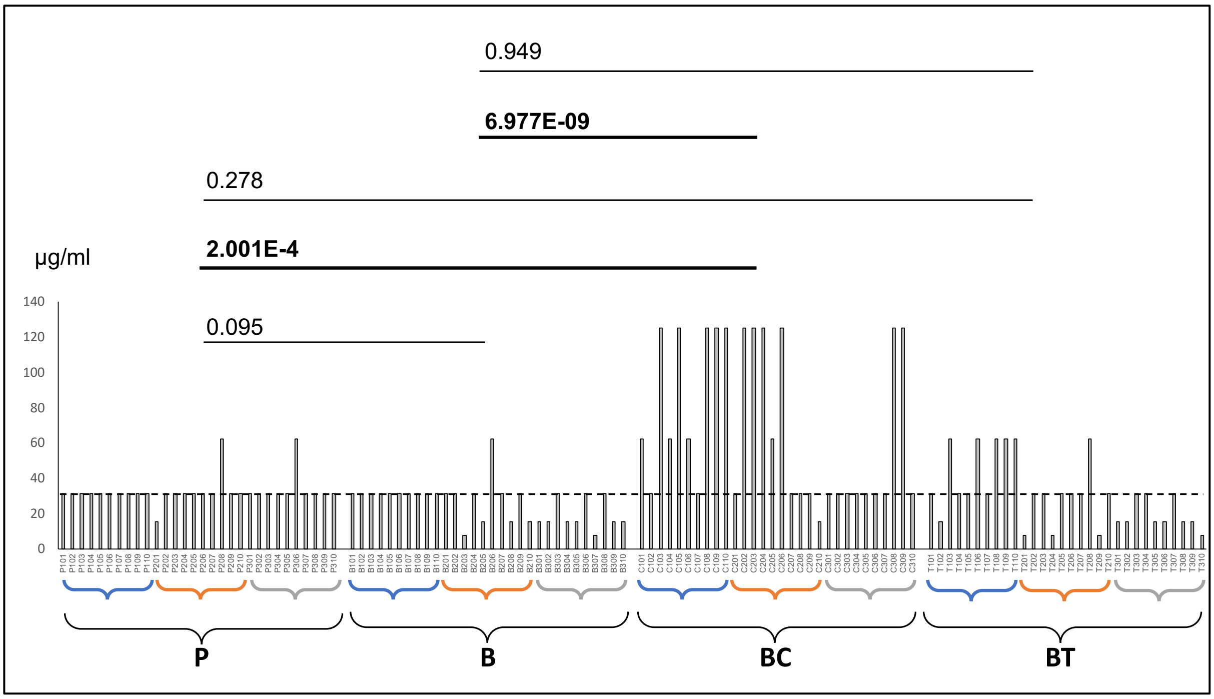

Supplementary Figure 1. Results of the erythromycin minimum inhibitory concentration (MIC) assay. X-axis: 30 planktonic isolates P101-P310 (P samples), 30 antibiotic-free biofilm effluent isolates B101-B310 (B samples), 30 ciprofloxacin-exposed biofilm effluent isolates C101-C310 (BC samples) and 30 tetracycline-exposed biofilm effluent isolates T101-T310 (BT samples). Each sample type includes 10 isolates from each biological replicate 1 (blue parentheses), biological replicate 2 (orange parentheses) and biological replicate 3 (gray parentheses). The height of gray bars represents the MIC levels of erythromycin (in µg/ml). Consensus MIC levels in the initial planktonic cultures are shown by the horizontal dashed line. P values denote differences between sample pairs based on nested mixed factor ANOVA test followed by Turkey HSD post-hoc test. P values showing statistically significant ($p < 0.05$) differences are presented in bold.

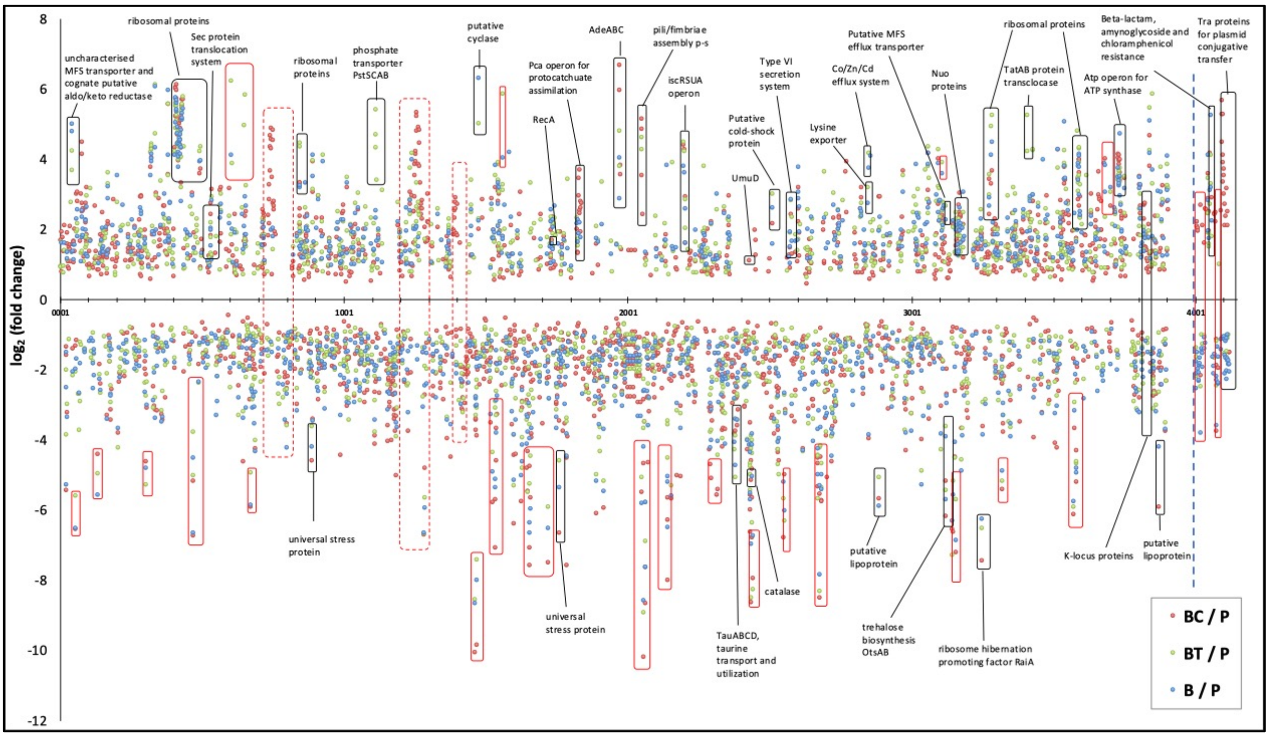

Supplementary Figure 2. Transcriptomic changes (log_2 fold change; p adj < 0.05) observed in ciprofloxacin- (orange circles) and tetracycline- (green circles) exposed biofilms and antibiotic-free biofilms (blue circles) compared to stationary phase planktonic cultures. The X-axis represents locus tag numbers on the AB5075-UW main chromosome and the largest plasmid 1 (separated by the blue dashed line). Genes with unknown functions are outlined by red rectangles, putative phage genes are outlined by red dashed rectangles. Three independent biological replicates were used for evaluating significance.

Supplementary Data 1. Mutations (≥ 50% frequency) detected in planktonic isolates (P101-P310), as well as in antibiotic-free biofilm (B101-B310), ciprofloxacin-exposed biofilm (C101-C310) and tetracycline-exposed biofilm effluent isolates (T101-T310). Mutation presence/absence is presented by numbers ≤ 1.0 denoting the predicted frequency where 1.0 represents 100% frequency.

Supplementary Data 2. Correlations between mutations, between mutations and the growth regime, between mutations and phenotypic measures, and between mutations and biological replicates.

Supplementary Data 3. RNA sequencing results showing differential expression of genes (p adj < 0.05) in biofilm samples: B (antibiotic-free biofilm), BC (ciprofloxacin-exposed biofilm), and BT (tetracycline-exposed biofilm) when compared to stationary phase planktonic cultures (/P); or in antibiotic-exposed samples BC and BT when compared to antibiotic-free biofilms (/B).
